# Do Genetic Diversity Effects Drive the Benefits Associated with Multiple Mating? A Test in a Marine Invertebrate

**DOI:** 10.1371/journal.pone.0006347

**Published:** 2009-08-12

**Authors:** Laura McLeod, Dustin J. Marshall

**Affiliations:** School of Biological Sciences, The University of Queensland, Brisbane, Australia; University of Exeter, United Kingdom

## Abstract

**Background:**

Mothers that mate with multiple males often produce higher quality offspring than mothers that mate with a single male. By engaging in polyandry, mothers may increase their chances of mating with a compatible male or promote sperm competition - both of which act to increase maternal fitness via the biasing of the paternity of offspring. Surprisingly, mating with multiple males, can carry benefits without biasing paternity and may be due simply to differences in genetic diversity between monandrous and polyandrous clutches but role of genetic diversity effects in driving the benefits of polyandry remains poorly tested. Disentangling indirect, genetic benefits from genetic diversity effects is challenging but crucial if we are to understand the selection pressures acting to promote polyandry.

**Methodology/Principal Findings:**

Here, we examine the post-fertilisation benefits of accessing the sperm of multiple males in an externally fertilising polychaete worm. Accessing the sperm of multiple males increases offspring performance but this benefit was driven entirely by genetic diversity effects and not by the biasing of paternity at fertilisation.

**Conclusions/Significance:**

Previous studies on polyandry should be interpreted cautiously as genetic diversity effects alone can explain the benefits of polyandry yet these diversity effects may be difficult to disentangle from other mechanisms. We suggest that future studies use a modified experimental design in order to discriminate between genetic diversity effects and indirect, genetic benefits.

## Introduction

Females typically mate with more than one male in a reproductive cycle [1] and in a wide range of organisms, females that have mated with multiple males enjoy higher reproductive success and/or produce higher quality young [2], [3], [4]. While females in some species gain direct benefits from polyandry in the form of nuptial gifts, territorial defence, or parental care, in many other species, the benefits of polyandry are thought to be indirect [5], [6].

Indirect, or genetic benefits of polyandry are thought to be driven by multiple, though non-mutually exclusive, mechanisms [6]. First, by mating multiply, females create a competitive arena whereby sperm from a range of males compete for the fertilisation of eggs. Instigating sperm competition can carry two benefits: good sperm may produce fitter offspring (the ‘good sperm-good genes’ hypothesis) or may produce sons that also have more competitive sperm themselves [sometimes termed the ‘sexy sons’ hypothesis 7], [8]. A second proposed mechanism for an indirect benefit of polyandry is that it allows mate choice to occur post-copulation. Whilst females of many species can bias paternity before copulation through the active choice of some males over others, females of other species bias paternity through the differential utilisation of sperm from some males over others [9]. Thus, by mating with multiple males, females may be able to ‘trade up’ to a higher quality, or a more compatible male if she encounters one while still ensuring that her eggs could be fertilised in the meantime [10]. The above hypotheses predict that multiple mating will only carry a benefit if paternity is biased towards a ‘better’ or more compatible male but recent studies suggest that mating with multiple males can increase offspring performance in the absence of any biases in paternity.

A number of recent studies have shown that mating with multiple males results in offspring with higher performance but without the expected biases towards better or more compatible males [11], [12], [13], [14]. How does mutliple mating increase offspring performance in the absence of biases in paternity? The answer may lie in the fact that clutches of offspring produced by mothers that have mated multiply have higher genetic diversity over all compared to clutches produced by mothers that have mated with a single male. Genetic diversity has repeatedly been suggested as a potential mechanism for driving the benefits of multiple mating [6], [15], [16]. Furthermore, recent ecological studies support the idea that genetic diversity should intrinsically affect offspring survival through ‘complementarity’ effects. In ecological studies, complementarity effects occur because genetically different individuals compete less intensely for limiting resources than genetically similar individuals and as such, the overall productivity of a genetically heterogeneous population is higher than that of a genetically homogenous population [17]. Indeed, it has been suggested that mutliple mating decreases competition among siblings (either *in utero* or *post partum*) by increasing genetic diversity within a clutch, thereby increasing overall offspring survival or performance [5], [18], [19], [20] However, unequivocally testing the suggestion that genetic diversity carries intrinsic benefits for offspring (and thus mothers) is difficult in organisms with internal fertilisation. In internal fertilisers, mothers can bias allocation to their offspring according to the paternity of those offspring [21], [22] and thus maternal effects in internal fertilisers could confuse the relative contributions of genetic diversity effects with other potential mechanisms that drive the benefits associated with multiple mating. Thus, the life-histories of internal fertilisers make it difficult to distinguish between the effects of genetic diversity and the other benefits of multiple mating that arise due to the biasing of paternity. Nevertheless, disentangling the competing mechanisms that could be driving the benefits of multiple mating is crucial if we are to understand the evolution of mechanisms that promote multiple mating. One way of avoiding the logistical problems posed by internal fertilisers is to instead examine the relative effects of multiple mating and genetic in organisms with external fertilisation [2], [23].

Here we use an externally fertilising species, where sperm and eggs are shed into the external environment, to examine whether genetic diversity affects offspring performance in the absence of biases in paternity. In external fertilisers, ejaculates and clutches of eggs can be divided up and multiple crosses among different males and females can be done simultaneously and repeatedly across developmental stages, avoiding the potentially confounding influence of ejaculate effects and maternal effects [4], [23]. External fertilisers therefore offer an excellent opportunity for elucidating the underlying mechanisms driving the benefits associated with multiple mating [24]. Although encouraging polyandry is predicted to increase the risk of polyspermy in broadcast spawners [25], it is not yet known whether encouraging multiple paternity carries a cost in this group more generally. In the absence of such knowledge, it might be argued that there are more compelling reasons to examine the benefits of polyandry in internal fertilisers for which costs of polyandry are more apparent. However, examining the benefits of multiple mating in external fertilisers *per se* is not the major goal of our study. Instead, we seek to take advantage of the life-history of external fertilisers to ask questions that are crucial to understanding the evolution of polyandry in general, but are difficult to test explicitly in internal fertilisers.

**Figure 1:**
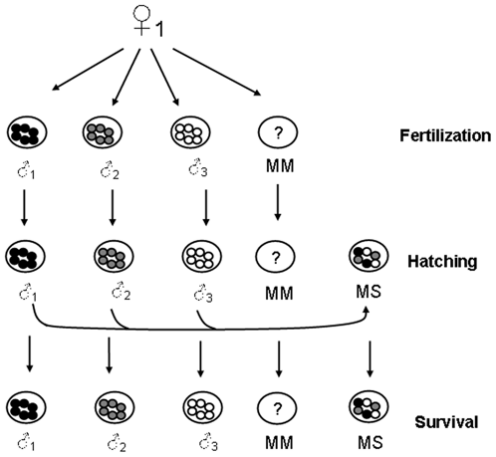
Experimental design for Experiment 2. As per experiment 1, offspring performance was measured at three life-history stages: fertilisation, hatching and larval survival. Two replicates were created for each of the three males separately (denoted as ♂1, ♂2, ♂3), a multiple males treatment (denoted as MM) and a mixed-singles treatment (denoted as MS). The mixed-singles treatment was created by taking the fertilised eggs of all three males (after the fertilisation rate had been calculated) and placing them together in a separate container. Therefore, the mixed-singles treatment gives an estimate of the hatching and post-hatching survival success of all three males developing together but in the absence of the potential for biasing paternity at fertilisation.

We investigated the post-fertilisation benefits of polyandry in a marine broadcast spawner, *Galeolaria caespitosa*. We first examined the post-fertilisation benefits of polyandry using previously published methods [26] and found that offspring from clutches sired by multiple males had higher hatching success and survival rates than clutches sired by those same males in isolation. We then used a new experimental approach (Figure 1) to determine if the post-fertilisation benefits of associated with accessing the sperm of multiple males were due to a biasing of paternity at fertilisation (as would be expected if there was a indirect, genetic benefit to polyandry) or due to an increase in genetic diversity post-fertilisation and found that genetic diversity effects explained the benefits associated with multiple mating entirely.

## Materials and Methods

### Study species

*Galeolaria caespitosa* is a sessile, serpulid polychaete worm common to temperate intertidal waters of south-eastern Australia. Individual worms are enclosed within calcareous tubes, and occur in mixed sex ‘colonies’ at a range of densities, from isolated individuals through to very dense aggregations. During spawning, reproductively mature adults release eggs and sperm, which, if fertilised, produce a pelagic, feeding (planktotrophic) larvae. Larval development proceeds through two characteristic stages: the free-swimming trochophore and the demersal nectochaete larvae [27].

### General methods – gamete collection and larval rearing

Whole colonies were taken from a population at Burleigh Heads (28°05′ S, 153°27′ E) and transported to the University of Queensland, St Lucia. Colonies were held in the laboratory in aerated tanks, containing seawater from the site of collection, at room temperature for up to a week. *Galeolaria caespitosa* produce viable gametes throughout the year and the sexes are easily distinguished upon removing the worms from their tubes by the colour the abdomen (Marsden and Anderson 1981). We collected gametes using standard techniques for this species [26]. To estimate sperm concentrations we did three to five replicate counts with an improved Nubauer haemocytometer. Sperm concentrations were kept constant within experiments by diluting sperm to a concentration of 4.4×10^6^ sperm/mL with filtered seawater. This concentration typically results in moderate to high levels of fertilisation success [26].

Eggs were incubated with the sperm solutions (total volume: 1 ml of solution) for 15 minutes at 22°C before being rinsed free of sperm on a 25 µm Nitex mesh and placed into a new polyethylene container with filtered seawater. Once the fertilisation rate of eggs had been obtained (see below) we placed the zygotes into a new 40 mL specimen jar with between 5 mL and 10 mL of seawater. The amount of seawater we used varied because we kept the ratio of developing embryos to seawater volume constant among jars. Importantly, we knew how many zygotes were in each jar. The zygotes were then left to develop for 20 hours at a constant temperature (22°C) before being assayed for hatching success (see below). After we estimated hatching success, we collected 36 trochophores and placed them into fresh containers with 25 mL of unfiltered seawater and allowed them to continue to develop at a constant temperature of 22°C. Our collection of larvae was haphazard rather than strictly random but our collection methods were identical among our different treatments throughout our experiments ensuring that no biases were introduced to our findings. For both of our estimates of post-fertilisation performance, larval density was constant across treatments and among vials.

### Assaying offspring traits

To estimate the fertilisation success of eggs, we examined the eggs 90 minutes after initial exposure to sperm under a dissecting microscope (Magnification: 40×). We classed eggs as ‘fertilised’ if they had begun regular cell divisions and ‘unfertilised’ if they showed no development whatsoever. This is a standard technique for assessing fertilisation success in this species [28] and after 90 minutes, fertilised eggs were between the 2-cell and 8-cell stage.

To estimate hatching success of the zygotes, we examined the larvae with a dissecting microscope (Magnification: 40×) and classed larvae as ‘hatched’ if they had reached the trochophore stage and ‘unhatched’ if they had disappeared (recall that we knew how many zygotes had been placed in each jar), were deformed or were yet to become trochophores. To estimate post-hatching survival after three days, we classed larvae as ‘alive’ if they were actively swimming or responded to stimulation from a pipette. Larvae were classed as ‘dead’ if they were missing or were motionless on the bottom of the jar and did not respond to any stimulation.

### Experiment I – Are there post-zygotic benefits of multiple paternity?

To explore the benefits of accessing the ejaculates of multiple males, we used a “North Carolina II – Polyandry” design [26], [29]. In this design, for each ‘block’ we split the clutches of three females' eggs and the ejaculates of three males so that each male×female combination is represented and replicated twice. In the ‘single male’ treatment, females' eggs were exposed to the sperm of one male individually, while in the ‘multiple male’ treatment, the eggs were exposed to an equal proportion of sperm from all three males. We then examined fertilisation success, hatching success and post-hatching survival. We repeated these experiments to yield a total of 8 blocks.

To analyse the effect of exposure to ejaculates from multiple males at fertilisation on fertilisation success, hatching success and post-hatching survival, we used a mixed model ANOVA where Treatment (multiple vs. single) was a fixed factor and Block was a random factor. We had first included Female(Block) as an additional, random factor but as this factor explained little variation, was not significant and so was omitted from the analysis [30].

### Experiment II – Are the post-zygotic benefits of multiple paternity due to genetic diversity?

We found that offspring derived from batches of eggs exposed to the sperm of multiple males had high fertilisation success, hatching success and survival. Thus, our next goal was to determine whether these benefits were due to a biasing of paternity fertilisation or due to higher genetic diversity during post-zygotic development. Our experimental approach utilised the same design as that of Experiment I (i.e. North Carolina II – Polyandry) but with an added treatment – ‘mixed-singles’ (see Figure 1). We used the mixed single treatment to decouple the possible benefits of biasing paternity at fertilisation (sperm competition or female choice), from any benefits that are due to a higher degree of genetic diversity during development alone (Figure 1). For the mixed-singles treatment, eggs were fertilised in isolation with a single male's sperm as in the normal single male treatment but after fertilisation, the eggs were rinsed of sperm and 12 eggs from each monandrous cross were pooled with eggs of the same female that had been fertilised by the other males in that block. Thus, there was no opportunity for paternity bias in the mixed-singles treatment with equal proportions of eggs sired by each male but there was a higher genetic diversity of developing embryos in the mixed single treatment (as in the multiple-male treatment). Importantly, the number of developing zygotes in all three treatments was kept identical.

To analyse the effect of the single male, mixed-singles and multiple-male treatments on hatching success and post-hatching survival, we used the same analysis as described for Experiment I. However, when this analysis found a significant effect of the treatment, we used incremental planned comparisons to identify where the three treatments levels significantly differed from each other [30 p. 197]. We first tested whether mixed-singles and multiple-males were significantly different – when we found no difference, we pooled these treatments and compared them to the single male treatment.

## Results

### Experiment I – Are there post-zygotic benefits of multiple paternity?

**Figure 2:**
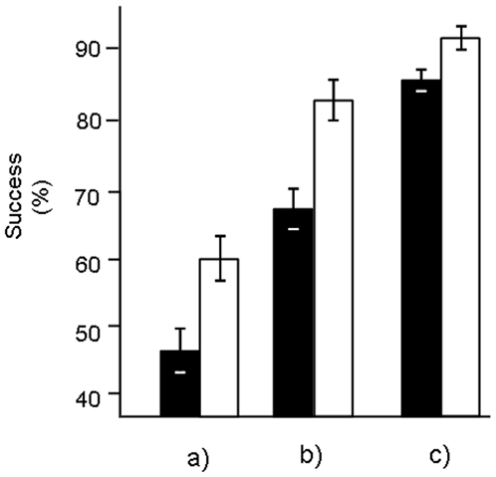
Effect of polyandry on a) fertilisation success; b) hatching success and c) larval survival in *Galeolaria caespitosa*. Solid bars and open bars show mean (±S.E.) for the single-males treatment and multiple-males treatment respectively.

Eggs that accessed the sperm of multiple males simultaneously achieved, on average, ∼17% higher rates of fertilisation than eggs that accessed sperm from the same males but in isolation (Treatment: F_1,9_ = 11.03, P = 0.009; Block: F_9,17_ = 11.93, P<0.001; Treatment×Block: F_9,17_ = 2.81, P = 0.032, Figure 2). These effects differed among experimental runs as revealed by a significant run×treatment interaction and when we explored this among run variation further, we found that the benefit of multiple males (i.e. the difference between the single male treatment and the multiple-male treatment) increased with the variation in fertilisation success among different males within individual blocks (F_1,26_ = 4.987, P = 0.034).

Batches that were fertilised by multiple males also had greater hatching success than eggs that were fertilised under single male conditions (Treatment: F_1,6_ = 10.65, P = 0.017; Block: F_6,12_ = 32.44, P<0.001; Treatment×Block: F_6,12_ = 1.86, P = 0.176). The analysis revealed no significant interaction between block and treatment, indicating that the benefits of accessing the sperm of multiple males on hatching were more consistent than at fertilisation. This post-zygotic benefit of multiple paternity also affected larval survival with larvae from the multiple-male treatment surviving better than larvae from the single male treatment (Treatment: F_1,6_ = 6.56, P = 0.043; Block: F_6,12_ = 9.51, P<0.001; Treatment×Block: F_6,12_ = 2.24, P = 0.104). On average, larval survival was 7% higher in larvae that were produced from mixed single matings compared to those from single male matings.

### Experiment II – Are the post-zygotic benefits of multiple paternity due to genetic diversity?

**Figure 3:**
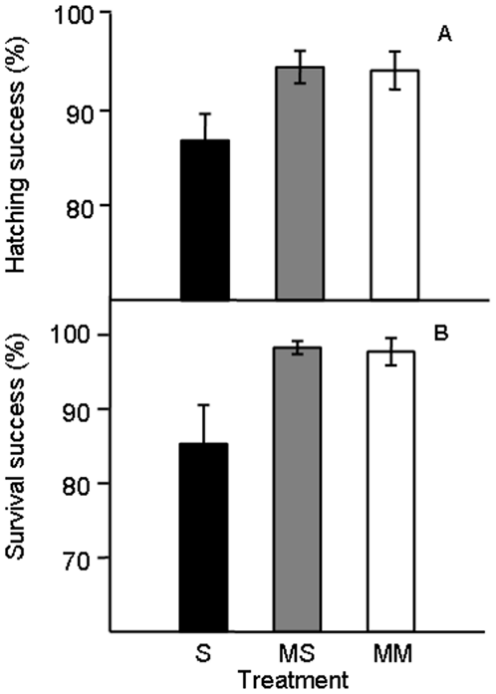
Mean (±S.E.) hatching success (Panel a) and larval survival (Panel b) of *Galeolaria caespitosa* offspring under single males (S), mixed-singles (MS) and multiple-males (MM) treatments.

Mean hatching success was significantly different among the different treatments (Treatment: F_2,5_ = 7.00, P = 0.003; Block: F_5,28_ = 2.54, P = 0.051; Treatment×Block: F_10,28_ = 0.91, P = 0.543 Figure 3). *Post-hoc* analysis revealed that this significant effect was driven largely by the lower hatching success in the single male treatment relative to the mixed-singles and multiple male treatments which, were almost identical (F_1,28_ = 0.009, P = 0.924; Figure 3). Overall, zygotes in the single male treatment hatched at significantly lower rates than the eggs in the mixed-singles and multiple male treatments combined (F_1,28_ = 13.988, P = 0.001).

When we examined larval survival, there was a significant interaction between Block and the treatment and so the main effect of treatment could not be examined in isolation (Treatment: F_2,4_ = 3.78, P = 0.120; Block: F_2,8_ = 0.64, P = 0.55 Treatment×Block: F_4,8_ = 3.89, P = 0.048). However, when we explored this interaction, the direction of the effect was consistent among blocks (i.e. the survival of mixed-singles and multiple male treatments was always greater than that of single male treatments) but the strength of the effect differed among blocks (Figure 3).

## Discussion

When eggs were exposed to the sperm of multiple males, we observed indirect benefits to those batches of eggs at every life history stage that we examined in *Galeolaria caespitosa*. Similar to other studies on broadcast spawners, eggs enjoyed higher fertilisation success when they were fertilised under polyandrous conditions [26], [29], [31], but in the current study, we found that these benefits persisted beyond fertilisation to affect hatching success and post-hatching survival. Based on our results, eggs that were fertilised by multiple males simultaneously have a 37% better chance of reaching the three-day-old larval stage than eggs that were fertilised by males in isolation. We also found strong block effects suggesting that the average genetic quality of males that were used in each block varied significantly from block to block. Our second round of experimental manipulations suggests that the benefits associated with accessing multiple ejaculates at fertilisation were not due to a biasing of paternity such that offspring from the best or most compatible males were overrepresented. Embryos produced under the mixed-singles treatment (where ‘choice’ at fertilisation was excluded but there was higher genetic diversity during development) had similar levels of larval performance to those produced under the multiple-male treatment. Thus it appears that the benefits associated with accessing the sperm of multiple males that we observed are due to an increased level of genetic diversity during embryonic and larval development alone.

While most reviews of polyandry benefits focus on good genes benefits or incompatibility avoidance [2], [4], [10], [23], [32], ours is not the first study to suggest that one of the benefits of polyandry is an increased level of genetic diversity in a brood of offspring. Studies on social insects in particular show fitness benefits of increased genetic diversity [33], [34]. More generally, increasing the genetic diversity of a brood of offspring may result in decreased sibling competition [35], [36], [37]. Similarly, recent ecological studies support the idea that competition will be reduced and productivity (in this case, survival) will be enhanced in populations where interacting individuals are genetically distinct from each other [17]. In our study, it may be genetic diversity facilitates niche partitioning with regards to the utilisation of resources (e.g. dissolved organic matter) but such effects require further testing [38]. Similarly, given that embryos sired by different males differ in their development rates [39], [40], periods of peak oxygen demand could be slightly offset through time in high diversity clutches. Studies on other broadcast spawners show that increasing embryo density during development decreases survival, and embryos sired by multiple males are better able to survive at higher densities than embryos sired by single males [29]. These findings suggest competition among embryos does occur and that a mixture of embryos mitigates the effects of competition [29]. Regardless of the exact mechanism that was responsible for the genetic diversity effects in our study, our findings have interesting implications for aquaculture operations – increasing the genetic diversity of larval cultures could increase yields of larvae over time (>30% in our study) – a previously unanticipated effect. The importance of genetic diversity effects observed in this study for field populations of *Galeolaria caespitosa* remains unknown. In some intertidal species that co-occur with *G. caespitosa*, eggs are spawned at low tide and development of embryos occurs at very high densities for at least one tidal cycle [41], [42], [43] but no field data specifically for *G. caespitosa* are available. We remain unsure about the applicability of our study to field populations of *G. caespitosa* but we suggest that our findings raise an important caveat for all studies that examine the benefits of polyandry.

Studies examining the benefits of multiple paternity in internal fertilisers usually manipulate sire number at mating and then keep progeny from different matings in family groups such that offspring from the same mother are often kept in the same containers or experimental area [reviewed in 2], [5], [44]. From an experimental design perspective, this seems appropriate given that the treatment (polyandry or monandry) is applied at the level of family and from a practical perspective, this avoids the onerous task of keeping every single offspring isolated. Thus, many polyandry studies may be inadvertently confounded: they cannot distinguish between the effects of genetic diversity and indirect genetic benefits. Consider that many studies of polyandry keep family groups in common containers where they must compete for any limiting resources [29], [45], [46], [47] – it is exactly these conditions that ecological theory predicts will result in the greatest competition when genetic diversity is low [17]. It is therefore unclear whether the benefits of polyandry so often observed in laboratory studies are due to indirect, genetic benefits or are simply due to increase in genetic diversity associated with polyandry reducing competition within family groups. There is a simple solution however: either pooling offspring from both polyandry and monandry treatments and tracking individuals in this pooled arena (this is ideal) or keeping individual offspring separated [e.g. 48] will avoid these problems (at least, if genetic diversity effects only carry a benefit *post partum*). We note that, in highly fecund species such as insects, rearing each offspring individually may not be practical but in such instances, pooling offspring both treatments and using genetic markers may represent a viable alternative. In mammals, where parental care is obligate (and genetic diversity effects could manifest during this stage), we recommend cross-fostering approaches to disentangle the effects of genetic diversity from indirect genetic benefits of polyandry. Previous studies on external fertilisers may also require reinterpretation in light of the results presented in this study. In three phyla of broadcast spawners, fertilisation success is higher in clutches of eggs fertilised by multiple males relative to clutches fertilised by males in isolation [26], [29], [31]. These studies interpreted the fertilisation benefits associated with polyandry as a compatibility effect but our results here suggest that these benefits could equally have been caused by genetic diversity effects on sperm performance during fertilisation.

Despite the ambiguity regarding the relative contribution of genetic diversity effects and indirect, genetic benefits, most studies and reviews of polyandry attribute the observed benefits of polyandry to indirect genetic benefits [5], [6], [44], [49], [50]. While several reviews acknowledge the potential role of genetic diversity [5], [6], [44], this mechanism is usually given less weight relative to other, indirect genetic benefits of polyandry. Given that recent studies find that polyandry in internal fertilisers can carry benefits in the absence of biases in paternity [such absences in paternity bias would not be predicted under a ‘good genes’ or ‘incompatibility avoidance’ scenario 11], [12], [13], we suggest that the potential role of genetic diversity in polyandry studies requires more attention.

## References

[1] Birkhead TR, Møller AP (1998). Sperm competition and sexual selection..

[2] Simmons LW (2001). The evolution of polyandry: an examination of the genetic incompatibility and good-sperm hypotheses.. Journal Evolutionary Biology.

[3] Zeh JA, Zeh DW (2001). Reproductive mode and the genetic benefits of polyandry.. Animal Behaviour.

[4] Simmons LW (2005). The evolution of polyandry: Sperm competition, sperm selection and offspring viability.. Annual Reviews of Ecology & Systematics.

[5] Jennions MD, Petrie M (2000). Why do females mate multiply? A review of the genetic benefits.. Biological Reviews.

[6] Neff BD, Pitcher TE (2005). Genetic quality and sexual selection: an integrated framework for good genes and compatible genes.. Molecular Ecology.

[7] Kokko H, Jennions MD, Brooks R (2006). Unifying and Testing Models of Sexual Selection.. Annual Review of Ecology, Evolution, and Systematics.

[8] Birkhead TR, Moller AP, Sutherland WJ (1993). Why Do Females Make It So Difficult for Males to Fertilize Their Eggs.. Journal of Theoretical Biology.

[9] Eberhard WG (1996). Female control: sexual selection by cryptic female choice..

[10] Neff BD, Pitcher TE (2005). Genetic quality and sexual selection: an integrated for good genes and compatible genes.. Molecular Ecology.

[11] Bilde T, Maklakov AA, Schilling N (2007). Inbreeding avoidance in spiders: evidence for rescue effect in fecundity of female spiders with outbreeding opportunity.. Journal of Evolutionary Biology.

[12] Teng ZQ, Kang L (2007). Egg-hatching benefits gained by polyandrous female locusts are not due to the fertilization advantage of nonsibling males.. Evolution.

[13] Zeh JA, Zeh DW (2006). Outbred embryos rescue inbred half-siblings in mixed-paternity broods of live-bearing females.. Nature.

[14] Dunn PO, Lifjeld JT, Whittingham LA (2009). Multiple paternity and offspring quality in tree swallows.. Behavioral Ecology and Sociobiology.

[15] Yasui Y (1998). The ‘genetic benefits’ of female multiple mating reconsidered.. Trends in Ecology & Evolution.

[16] Arnqvist G (1989). Multiple Mating in a Water Strider - Mutual Benefits or Intersexual Conflict.. Animal Behaviour.

[17] Hughes AR, Inouye BD, Johnson MTJ, Underwood N, Vellend M (2008). Ecological consequences of genetic diversity.. Ecology Letters.

[18] Oldroyd BP, Rinderer TE, Harbo JR, Buco SM (1992). Effects of Intracolonial Genetic Diversity on Honey-Bee (Hymenoptera, Apidae) Colony Performance.. Annals of the Entomological Society of America.

[19] Ridley M (1993). Clutch Size and Mating Frequency in Parasitic Hymenoptera.. American Naturalist.

[20] Loman J, Madsen T, Hakansson T (1988). Increased Fitness from Multiple Matings, and Genetic-Heterogeneity - a Model of a Possible Mechanism.. Oikos.

[21] Cunningham EJA, Russell AF (2000). Egg investment is influenced by male attractiveness in the mallard.. Nature.

[22] Evans JP, Zane L, Francescato S, Pilastro A (2003). Directional postcopulatory sexual selection revealed by artificial insemination.. Nature.

[23] Ivy TM (2007). Good genes, genetic compatibility and the evolution of polyandry: use of the diallel cross to address competing hypotheses.. Journal of Evolutionary Biology.

[24] Dziminski MA, Roberts JD, Simmons LW (2008). Fitness consequences of parental compatibility in the frog *Crinia georgiana*.. Evolution.

[25] Bode M, Marshall DJ (2007). The quick and the dead? Sperm competition and sexual conflict in the sea.. Evolution.

[26] Marshall DJ, Evans JP (2005). The benefits of polyandry in the free-spawning polychaete *Galeolaria caespitosa*.. J Evol Biol.

[27] Marsden JR, Anderson DT (1981). Larval development and metamorphosis of the serpulid polychaete *Galeolaria caespitosa* Lamark.. Aust J Mar Freshwater Res.

[28] Marshall DJ, Evans JP (2005). Does egg competition occur in marine broadcast spawners?. J Evol Biol.

[29] Marshall DJ, Evans JP (2007). Context dependent genetic benefits of polyandry in a marine hermaphrodite.. Biology Letters.

[30] Quinn GP, Keough MJ (2002). Experimental design and data analysis for biologists..

[31] Evans JP, Marshall DJ (2005). Male x female interactions influence fertilisation success and mediate the benefits of polyandry in the sea urchin *Heliocidaris erythrogramma*.. Evolution.

[32] Zeh JA, Zeh DW (2003). Toward a new sexual selection paradigm: Polyandry, conflict and incompatibility (Invited article).. Ethology.

[33] Hughes WOH, Boomsma JJ (2004). Genetic diversity and disease resistance in leaf-cutting ant societies.. Evolution.

[34] Mattila HR, Seeley TD (2007). Genetic diversity in honey bee colonies enhances productivity and fitness.. Science.

[35] Griffiths SW, Armstrong JD (2001). The benefits of genetic diversity outweigh those of kin association in a territorial animal.. Proceedings of the Royal Society of London Series B-Biological Sciences.

[36] Barton NH, Post RJ (1986). Sibling Competition and the Advantage of Mixed Families.. Journal of Theoretical Biology.

[37] Forsman A, Ahnesjo J, Caesar S (2007). Fitness benefits of diverse offspring in pygmy grasshoppers.. Evolutionary Ecology Research.

[38] Manahan DT (1990). Adaptations by invertebrate larvae for nutrient acquisition from seawater.. American Zoologist.

[39] Evans JP, Garcia-Gonzalez F, Marshall DJ (2007). Sources of genetic and phenotypic variation in sperm performance and larval traits in a sea urchin.. Evolution.

[40] Marshall DJ, Bolton TF (2007). Effects of egg size on the development time of non-feeding larvae.. Biological Bulletin.

[41] Marshall DJ (2002). In situ measures of spawning synchrony and fertilization success in an intertidal, free-spawning invertebrate.. Marine Ecology Progress Series.

[42] Marshall DJ, Semmens D, Cook C (2004). Consequences of spawning at low tide: limited gamete dispersal for a rockpool anemone.. Marine Ecology Progress Series.

[43] Castilla JC, Manriquez PH, Delgado AP, Gargallo L, Leiva A (2007). Bio-foam enhances larval retention in a free-spawning marine tunicate.. Proceedings of the National Academy of Sciences of the United States of America.

[44] Simmons LW (2005). The evolution of polyandry: Sperm competition, sperm selection, and offspring viability.. Annual Review of Ecology Evolution and Systematics.

[45] Klemme I, Ylonen H, Eccard JA (2008). Long-term fitness benefits of polyandry in a small mammal, the bank vole *Clethrionomys glareolus*.. Proceedings of the Royal Society B.

[46] Pai A, Feil S, Yan G (2007). Variation in polyandry and its fitness consequences among populations of the red flour beetle, *Tribolium castaneum*.. Evolutionary Ecology.

[47] Dunn DW, Sumner JP, Goulson D (2005). The benefits of multiple mating to female seaweed flies, *Coelopa frigida* (Diptera: Coelpidae).. Behavioral Ecology and Sociobiology.

[48] Maklakov AA, Lubin Y (2006). Indirect genetic benefits of polyandry in a spider with direct costs of mating.. Behavioral Ecology and Sociobiology.

[49] Tregenza T, Wedell N (1998). Benefits of multiple mates in the cricket *Gryllus bimaculatus*.. Evolution.

[50] Zeh JA, Zeh DW (1997). The evolution of polyandry.2. Post-copulatory defences against genetic incompatibility.. Proceedings of the Royal Society of London Series B.

